# Larval predation in malaria vectors and its potential implication in malaria transmission: an overlooked ecosystem service?

**DOI:** 10.1186/s13071-019-3479-7

**Published:** 2019-05-08

**Authors:** Olivier Roux, Vincent Robert

**Affiliations:** 1 MIVEGEC Unit, IRD-CNRS, Université de Montpellier, Montpellier, France; 2 Institut de Recherche des Sciences de la Santé (IRSS), 01 BP 545, Bobo-Dioulasso 01, Burkina Faso

**Keywords:** *Anopheles*, Biological control, Carry-over effect, Environmental stress, Larval source management, Life-history trait, Non-consumptive effect, Oviposition, Vectorial capacity

## Abstract

The role of aquatic predators in controlling the anopheline aquatic stage has been known for decades. Recently, studies have highlighted that exposition to predation stress during aquatic development can have a profound impact on life-history traits (e.g. growth rate, fecundity and longevity) and consequently on the ability of adults to transmit human malaria parasites. In this study, we present a review aiming to contextualize the role of *Anopheles* larvae predators as an ecosystem factor interacting with the malaria pathogen through its vector, i.e. the female adult *Anopheles*. We first envisage the predator diversity that anopheline vectors are susceptible to encounter in their aquatic habitats. We then focus on mosquito-predator interactions with a special mention to anti-predator behaviors and prey adaptations developed to deal with the predation threat. Next, we address the direct and indirect effects of larval predation stress on mosquito populations and on individual life-history traits, which strongly suggest some carry-over effect of the impact of larval predation on vectorial capacity. The last part addresses the impact of human activities on larval predation. Concluding remarks highlight gaps in the knowledge of anopheline bio-ecology which may constitute avenues for researchers in the future.

## Background

The productivity of larval habitats is a key factor which governs the size of mosquito populations and, in the case of anopheline vectors, the transmission of human malaria parasites. Like most short-term living organisms, anopheline mosquitoes present huge variations of adult density, depending on the more or less suitable conditions prevailing in larval habitats. Among the adverse conditions, predation is of primary importance. In his seminal paper published in 1958 “Predation on larvae of *Anopheles gambiae* Giles”, M. Christie exposes a series of experimental studies before and after removing the natural fauna of a semi-permanent pool and gives a convincing demonstration of the great extent of predation [1].

Malaria infections are a threat to about half of the worldʼs population and are estimated to be responsible for more than 435,000 deaths in 2017, mainly in sub-Saharan Africa [2]. Long-lasting insecticide-treated nets are widely distributed and indoor residual spraying is recommended to limit human-vector contacts [2]. Rapid diagnostic tests are now available and bi-therapy drug administration is used to cure infection in patients. However, these methods are continuously challenged by the emergence and spread of both insecticide and drug resistances in the vectors and pathogens, respectively [2]. Unfortunately, because the blood-feeding habits of mosquitoes are a key behavior in the transmission process of malaria, and because it was thought that mosquitoes could be controlled with insecticides, other basic life-history traits, behaviors and ecologies remain deeply unexplored [3, 4]. Even though alternative control strategies are being investigated, they are still not efficient enough to lead to a long-term malaria control at moderate costs.

Five species of *Plasmodium*, the pathogens responsible for malaria, are transmitted to humans by the bites of infected female *Anopheles* mosquitoes. To date, among the 488 species of *Anopheles* recognized as valid [5], about 60 are capable of transmitting *Plasmodium* to humans. Although *Anopheles* mosquitoes are cosmopolite and able to colonize various environments, most of these species occupy a specific niche. At a regional scale, *Anopheles* distribution is dictated by the larval aquatic stages, the most demanding in terms of biotic and abiotic conditions within the species. As larvae have a poor capacity to disperse, they often have to face challenging environments and grow under high stress levels. A growing body of literature indicates that environmental stresses (e.g. competition, predation or food shortage) experienced during larval development can have a profound impact on life-history traits (e.g. growth rate, fecundity and longevity) [6–9] which are important factors in pathogen transmission through changes in adult vectorial capacity that measures the transmission potential of a given infectious agent by a vector population [10–13].

Disease ecologists are increasingly realizing that species interactions influence the intensity of epidemics in wildlife populations [14, 15]. As previously noted, the importance of predation of anophelines at aquatic stages has been known for decades, but renewed approaches shed light on the possible role of predation in disease dynamics demonstrating that predators can indirectly affect pathogen transmission through changes in host abundance (i.e. density-mediated effects) and through non-consumptive effects by altering the preyʼs phenotype and life-history traits. Predator-induced phenotypic changes can be especially pervasive in prey with discrete larval and adult stages for which exposure to biotic or abiotic stress during larval development can have strong carry-over effects on adult phenotypes [16, 17].

The role that predatory aquatic insects play as biocontrol agents in the natural regulation of larval and adult populations of mosquitoes has been known for a long time. However, the role of predators in pathogen transmission in mosquito-pathogen vectorial systems is overlooked. In this review, we contextualized the role of *Anopheles* larvae predators as an ecosystem factor interacting with the malaria pathogen through its vector, i.e. the female adult *Anopheles*. First, we briefly review the predator diversity that malaria vectors are susceptible to encounter in their aquatic habitats and describe their different specificities and efficiency at killing mosquito larvae. This part does not pretend to draw up an exhaustive list of all mosquito predators, nor of their complete characteristics, therefore in this regard, we suggest that the reader refers to the reviews cited in this section. The second part focuses on mosquito-predator interactions with a special mention to anti-predator behavior and prey adaptations. We then address the direct and indirect effects of larval predation on mosquito populations and on individual life-history traits, which lead to focus on the impact of larval predation stress on vectorial capacity. Finally, the last part addresses the impact of human activities on larval predation.

## Diversity of aquatic predators of anophelines

Aquatic larval environments are highly challenging. Mosquito larval mortality from eggs to adults has been estimated to be over 90% [18, 19]. Mortality attributable to aquatic predators is highly variable ranging from 19 to 54% in rice fields in Thailand and up to 96% in Kenya [19, 20], and from 2% to 96% in a semi-field experiment in Kenya [21] depending on the environment, predator species and their density.

Aquatic communities, even in small ponds, are complex and include many organisms belonging to many taxa. Disentangling this diversity and defining what organisms are predators of *Anopheles* larvae can be done by direct behavioral observations, visual examination of gut contents after dissection or by the means of electrophoretic, immunological and molecular methods [18, 22–27]. Several excellent annotated lists or research works have described in detail the community of mosquito predators in general, including both terrestrial and aquatic predators [23, 28, 29], as well as mosquito aquatic predators only [30–33] and more precisely, predators of *Anopheles* larvae [18, 22, 24, 34]. Most of these studies focused on rice fields or ponds but data on mosquitoes that thrive in forests (on ground level, in phytotelmata, in tree-holes, etc.) or in rivers or streams are scarce. Nevertheless, these studies revealed that the mosquito larvae predator community is mainly composed of aquatic vertebrates, arthropods and crustaceans.

Among the vertebrates, some amphibians are susceptible to be *Anopheles* larvae predators. Some tadpoles have been described to be associated to *Anopheles* aquatic habitats, but few of them have been identified as predators [18, 22, 24, 28, 35] and their appetite for *Anopheles* larvae is limited [20, 36]. Fishes, however, have been extensively studied both in the laboratory and in the field for their ability to eat mosquito larvae and their use as mosquito biological control agents [31]. Among the large range of larvivorous fishes (see [31] for a review) some are particularly efficient at controlling *Anopheles* larvae in many types of reservoirs. The most well-known fishes which are predators of *Anopheles* larvae, are *Gambusia affinis* (the mosquito fish) and *Poecilia reticulata* (guppies) which have been extensively used all over the world in anti-malarial programs to control mosquito populations in different kinds of reservoirs. *Gambusia affinis* originated from the coast of the western USA but are able to thrive in a large range of water quality i.e. freshwater, brackish water and salt marshes, from 0 °C to 45 °C, clear to turbid water, polluted urban water and in low dissolved oxygen concentration. They are sensitive to pesticides but are able to develop resistances [37, 38]. These large reaction norms to different abiotic conditions make *G. affinis* ideal for introduction in many parts of the world making this species the most widespread freshwater fish in the world [38, 39]. *Poecilia reticulata* is less flexible but has a tropical origin and consequently is adequate for use in malaria endemic areas. Both have shown high capacities to reduce the number of *Anopheles* larvae in laboratory and diverse aquatic habitats in the field, sometimes virtually clearing some villages from malaria cases for several years (but see [39–43]). However, the voracity of these two species, their ability to prey on almost any animal smaller than them without a real preference for mosquito larvae in the wild and their invasive nature gave rise to some concerns regarding negative environmental impacts [38, 39, 44]. Other fish species reported in Chandra et al. [31], for example *Oryzias melastigma* and *Danio rerio*, lowered the density of larvae and pupae of 76.2% and 86.8% in 6 days respectively and up to 100% in 12 days in rice fields. In barrels and containers, *Aphanius dispar* effectively reduced the breeding of *An. arabiensis* and *An. gambiae* by 97% in an urban area in Djibouti [31]. Another study has shown that the larval population of *An. stephensi* was reduced by 75% along the Goa coastal belt by *Aplocheilus blocki* [45]. Finally, it should be noted that the efficiency of fishes for really reducing malarial transmission, as predators of larval anophelines, is established in some cases but unproven in others [39, 42, 46].

Aquatic invertebrates are also very effective at killing mosquito larvae. They are more ubiquitous than fishes in relation to mosquito habitat types because of their ecological diversity, small size, short generation time, and, for insects, their ability for aerial colonization. Although preference for mosquitoes is common, aquatic invertebrate predators are polyphagous. Many of them are also rather generalist in their habitat selection [29]. In insects, several works have shown that the Hemiptera [with Notonectidae (backswimmers), Corixidae (water boatmans) and Nepidae (water scorpions)], the Coleoptera [with Hydrophilidae, Dytiscidae (water beetles)] and the Odonata (with dragonflies and damselflies) were the 3 main predator orders associated with *Anopheles* [1, 18, 47–53]. Their abundance is generally higher in permanent reservoirs [53] where they are less exposed than in temporary reservoirs which dry up. Their efficiency is highly variable depending on the habitat type, mosquito density and predator assemblage [30]. Some larvae of mosquito species are also larvivorous, the most efficient and the most studied being *Toxorhynchites* species (reviewed in [54]). These mosquitoes are cosmopolitan under the tropics and mainly inhabit tropical and subtropical forests. Females do not feed on blood but on plant liquid sources only, rich in carbohydrates. During their larval development they are able to feed upon 300–5000 mosquito larvae [55, 56] and exhibit a prepupal killing behavior during which they kill mosquito larvae without consuming them, including conspecific mosquitoes. They are resistant to starvation and fourth-instar larvae are able to stop their development during the dry season to emerge as adults during the first rains [57]. As they are more or less specialized in mosquito larvae feeding, females tend to search for water reservoirs with mosquito larvae and thus are able to disperse and to search for pools of water which are difficult to access for humans. However, they have a preference for very small shaded containers (tree-holes, tires, etc.) and thus preferentially prey upon *Aedes* species [58, 59] which in turn means they are poorly adapted for the control of *Anopheles* species in open environments even if some successes have been recorded [60]. Species of other mosquito genera such as *Lutzia*, *Culiseta*, *Aedes* and *Culex* present an intra-guild predation behavior toward other mosquito larvae, generally inhabit ground pools and can show some preferences for *Anopheles* larvae [29, 30, 61, 62]. Even cases of cannibalism of fourth-instar larvae on first-instar larvae have been observed [25].

Among crustacean, many copepods from the order Cyclopoida are known to prey upon mosquito larvae. Despite their small size (0.5–1.5 mm), they are able to attack prey twice their size. Although their consumption of mosquito larvae per copepod is moderate, they are very efficient at controlling mosquito populations because of their high reproductive capacity. Most of the cyclopoids are generalist predators but they can show a preference for *Anopheles* over *Culex* and *Cladoceran* [63]. They are virtually everywhere, from ground pool, rain-filled tires to bromeliad reservoirs [32, 64, 65] and can have access to larvae that usually escape from other predators in thick aquatic vegetation because of their tiny size. They also have the ability to resist to poor environmental conditions by entering dormancy (quiescence or diapause) and some species are able to resist the dryness of their reservoirs for months [66]. Copepods have been used to suppress *Aedes* populations in many villages in Asia and are also efficient at suppressing *Anopheles* populations [32]. Although their use did not show any environmental problems, they can represent a health risk in the few areas where Guinea worms (*Dracunculus medinensis*) remain as they are known to be an intermediate host of this parasite even though it is now almost eradicated [32].

Finally, most of the identified predators consume many other insect species and there is no evidence that any species preys exclusively on any of the anopheline mosquitoes [67]. The presence of a variety of predators in the aquatic habitats of anophelines is a necessary but insufficient condition of predation. Indeed, up to a certain level, some of the larvae succeed in surviving despite the presence of predators.

## How do mosquito larvae deal with predators?

Predator-prey interactions are driven by an arm race in which the two protagonists attempt to develop strategies to survive. Predators endeavor to detect, recognize, catch and consume their prey and prey to detect, recognize and escape their predators. Consequently, predator-prey relationships play a central role in species evolution, ecology and community dynamics [68]. Here, we will focus on the prey perspective by exploring the strategies developed by mosquitoes, the consequences on their biology and on malaria transmission.

Once a predator detects a prey, the later has two options: to fight or to flee. Mosquito larvae and pupae have little choice between these options because their ability to fight is presumably null and their abilities to flee are limited, especially in small water bodies in which few or no places are safe from predators. Indeed, if most prey can leave their habitat when the predation threat is too high, mosquito larvae are often stuck in the water reservoir into which they have been laid (with the exception of species breeding in running water). Nevertheless, if mosquito larvae cannot fight nor flee for long, they are not sitting ducks waiting for predators either. In this line, mosquitoes have developed behavioral strategies to limit predation risk at both oviposition site selection and larval development levels.

For blood-feeding mosquitoes, the choice of an oviposition site is a critical step in the realization of individual fitness as well as in population dynamics and consequently in disease transmission [69]. As mosquito larvae are restricted in their movement, a bad choice in oviposition site by the females will deeply affect progeny survivorship. Consequently, females have developed a series of behaviors, strategies and senses to detect the best sites to lay their eggs [69]. The predation risk is among the characteristics gauged by female mosquitos to select an oviposition site. Chemical cues can be used either at distance or by direct contact with the water of the site [69–71]. The ability to detect water presenting a predation threat and avoid it for oviposition is common but not ubiquitous in mosquitoes (see [72] for a review). For example, *G. affinis* (fish) and *Notonecta maculata* (backswimmer) odors deter oviposition by *Culex* species and *Culiseta longiareolata*, respectively [71, 73–75]. *Culiseta longiareolata* also reacts to the physical presence of *Anax imperator* larvae (dragonfly) [76]. Oviposition response is frequently conditioned by the common evolutionary history between prey and their predators. Logically, preys that are not used to encountering a given predator generally display a low antipredator behavior [74, 77]. However, in *Culex restuans*, females did not react equally to three different species of predatory fishes that their progeny is susceptible to encounter and which represent the same level of threat to larvae [78]. In *Culex pervigilans*, females are able to detect larval predator *Anisops wakefieldi* (backswimmer) meaning that factors other than the common evolutionary history can be implied [79]. This is for example the case in the study by Blaustein et al. [75] in which the *Chironomus riparius* females (midge) do not detect *N. maculata* (backswimmer). It can be explained by the respective ecology of both the prey and the predator. Indeed, *Chironomus* larvae live at the bottom of the water reservoir and are protected by mud chimney, while backswimmers prey at the surface or in the water volume. Consequently, encounter probabilities are low, and the backswimmer is not a threat to the larvae. Sometimes, females are also able to detect the intensity of the threat level and lay their eggs in the least risky reservoirs [80].

In *Anopheles* species, few studies have been conducted on oviposition behavior related to predation risk. Nevertheless, Munga et al. [81] have shown that *An. gambiae* females were able to detect *N. maculata* on chemical bases and laid less eggs in presence of the predator. However, *An. gambiae* females did not use the same chemical compounds than *C. longiareolata* to gauge the predation risk [82]. *Anopheles gambiae* females are also able to detect fishes such as *G. affinis* and *Carassius auratus* [83].

As some predators are mobile from one aquatic habitat to another, the threat level gauged by ovipositing females is not necessarily the one larvae will experiment during their entire development. Consequently, larvae have also developed adaptations to escape predation threat or to minimize predation risk. In mosquitoes, adaptations are mainly behavioral with larvae tending to adopt a less conspicuous behavior in presence of a threat [84–88]. In *Anopheles gambiae* (*s.l*.), a surface feeder which spends most of its time filtering for food at the water-air interface in the middle of the aquatic habitat, the detection of a predation risk (*Anisops jaczewskii*: Hemiptera, Notonectidae) induces a shift in behavior leading to a less active behavior (resting and feeding less) at the edges of the aquatic habitat ([86, 87] but see [89]). The predation threat can be detected through different cues depending on the species [90, 91]. In the *An. gambiae* complex, *An. coluzzii*, *An. gambiae* (*s.s.*) and *An. arabiensis* use chemical cues issued from a predation act and particularly cues originating from killed and pre-digested larvae. *Anopheles arabiensis* and *An. gambiae* (*s.s.*) also use physical cues such as predator vibrations [87]. However, the three species are sensitive to different chemical cue concentrations reflecting different threat sensitivities related to their respective ecology [92]. Indeed, *An. coluzzii* mainly thrives in permanent reservoirs with a high predator density and many other organisms [53]. In this case, vibrations are certainly a poor information on the risk level and a too strong reaction to chemical cues could hinder the larval growth in an excessive way. Consequently, *An. coluzzii* larvae developed a gradual anti-predator response finely-tuned to the risk level to adjust the best trade-off between feeding and anti-predator behavior to minimize the cost of this anti-predator behavior (see below). In temporary reservoirs, shared by both *An. gambiae* and *An. arabiensis*, predators are less numerous, and the temporality of the water reservoir can induce frequent change in predator density. Consequently, as predators come and go, the presence of chemical cues is not necessarily instructive on the current risk level and physical cues, such as predator born-vibrations, could be used as an ultimate confirmation of the threat before triggering any anti-predator response [87]. These anti-predator behaviors allow prey to survive; however, such investments in anti-predator behavior have a cost and lead to direct and indirect impacts on individual life-history traits of the mosquito.

## Impact of larval predation stress on mosquito life-history traits and malaria transmission

If prey-predator interactions were mainly broached from the prey consumption angle to determine their direct and indirect effects on prey density, recently, non-consumptive effects of predators on prey has been considered [93, 94]. It is now recognized that non-consumptive effects can be greater than consumptive effects [93, 95, 96]. Indeed, when under predation threat, individuals may adopt different resource allocation strategies to favor their direct survival to the detriment of growth, reproduction and self-maintenance [97]. Energy or time invested in a trait or a task cannot be invested in another one. Consequently, these trade-offs have a cost on different prey life-history traits, individual fitness and *in fine* on prey population dynamics [94, 97].

In mosquito larvae, including *Anopheles* species, the main defense line is behavioral. Faced with a predation risk, larvae adopt a less conspicuous behavior spending more of their time resting and less time feeding [84–88, 98]. According to the threat level, larvae adopt a trade-off between these two behaviors: the higher the threat level is, the lower the larval feeding rate is [92, 99, 100]. However, this trade-off has a cost on both larval development and survival as well as a non-intuitive carry-over effect on adult life-history traits [6, 8, 12, 83, 101, 102]. Indeed, in *An. coluzzii* larvae exposed to the mere presence of backswimmers or fishes, the induced stress reduces larval survivorship and increases development duration [12, 83]. In adults, mosquito size, fecundity and survivorship are also negatively affected [12] as in other mosquito species [6, 8, 101, 102].

Lower food intake is probably not the only reason for the negative impact on life-history traits. Indeed, while under predation threats, prey are expected to allocate their resources to potential “fight-or-flight” situations and consequently convert less nutrients into body mass or reproduction (mainly proteins) to the benefit of carbohydrates in order to increase their metabolic rates (i.e. respiration) to fuel emergency functions [103–106]. Altogether, these physiological adaptations have effects on resource budgets, oxidative status, and thus long term life-history traits [103, 107–110]. Recently, predation stress has been shown to be a source of oxidative stress [107] affecting both enzymatic defenses [111–114] and life-history traits such as longevity, fecundity and mobility [115, 116]. Indeed, an increased metabolic rate is associated to increased respiration [106, 117]. If this physiological change is beneficial to drive survival in a life-threatening situation, it may affect long-term body maintenance. Indeed, a higher oxygen consumption generates a higher level of reactive oxygen species which, if unbalanced by an anti-oxidant machinery, can lead to oxidative stress resulting in cumulative damages to lipids, proteins and DNA [109, 110, 118]. Damages to these molecules affect the structure and functioning of membranes, disturb signal transduction, reduce muscle efficiency, induce changes in secondary and tertiary structures of proteins jeopardizing enzyme functioning, induce base mutations with consequences on phenotypes and also shorten telomeres accelerating cell senescence ([118] and references therein). Altogether, oxidative challenges and damages have a cost on resources, cell homeostasis and body maintenance. However, oxidative stress induced by predation stress in mosquitoes has never been investigated and deserves more attention. All the life-history trait alterations mentioned above have effects beyond mosquito fitness alone. When the *Anopheles* are considered for their function as disease vectors, the reduction of their densities (through their direct consumption, larval mortality or fecundity reduction), the alteration of their energy budget, the reduction of their size and longevity are all important factors of their susceptibility to be infected by pathogens and their capacity to transmit these pathogens. Indeed, it has been shown that different larval stresses such as competition, parasitism or diet could reduce mosquito susceptibility to pathogens. For example, in *Aedes* sp., susceptibility to viruses (La Cross, dengue and Sindbis) was reduced when exposed to an inter- or intra-specific competition during the larval stage [119–121]. In *Anopheles* sp., susceptibility to *Plasmodium* sp. was also altered when previously infected with microsporidies [122–126] or exposed to food deprivation [13]. However, Roux et al. [12] failed to highlight any effect of larval predation stress on susceptibility to *P. falciparum* in *An. coluzzii*. Nevertheless, using an epidemiological model, Roux et al. [12] showed that the life-history trait alteration by larval predation stress should overall decrease malaria transmission mainly because of both lower fecundity and longevity which are important factors of the mosquitoes’ vectorial capacity.

The mere presence of predators can also impact malaria epidemiology through oviposition behavior with females searching for aquatic habitats free of predators, thus impacting adult distribution, blood-meal frequency and survival. Indeed, females in search for adequate aquatic habitats are supposed to spend more time flying between aquatic habitats and blood-meal locations, increasing both oxidative stress and risk of aerial predation, reducing their energy budget and potentially taking fewer blood meals in their life ([72] and references therein; [127]). Altogether, larval predation shows complex effects on mosquitoes and pathogen transmission.

## How can human activities or interventions affect larval predation?

The aim of larval source management is to modify mosquito aquatic habitats to reduce mosquito populations. It can be done through (i) the spread of chemical or biological larvicides; (ii) permanent habitat modifications (i.e. drainage); (iii) temporary habitat manipulations (i.e. flushing, shading, etc.); and (iv) the release of biological control agents (parasites or predators) (all reviewed in [128]). These actions can be done on purpose or for other goals but in all cases, they must be done with caution. Indeed, a poor management of such actions can lead to a reduction of predator pressures exerted on prey through the diminution of predator densities or by impeding predator movements. The consequence is a reduction of prey-predator interactions which leads to the disruption of ecosystem services provided by predators.

The most obvious way human activities or interventions impact larval predation effects is through the use of chemical pesticides and the presence of pollutants in water which affect aquatic biodiversity [129, 130]. As chemical insecticides also kill competitors and predators, and because the mosquito population is more prone to recover than their predators and to develop resistances, indirect effects of insecticides on mosquito larvae can be counter-productive. Indeed, it has been reported that short term mosquito density can be increased by the use of insecticides because of the reduction of predator density [67, 131–133].

Land management such as land leveling to reduce the number of puddles, field draining, wetland destruction or management of river banks and lake shores have been a popular way to control mosquito aquatic habitats [134, 135]. However, these methods reduce the structural complexity of the habitats and increase their isolation. Reduction in habitat structural complexity induces fewer shelters for both prey and predators. However, the absence of shelters increases intraguild predation (predator-predator interactions) which reduces predation pressure on prey [136, 137]. The elimination of all mosquito aquatic habitats is difficult [138], thus land management tends to increase habitat isolation which is not a problem for mosquitoes which are good at dispersing. Moreover, because predators have lower local densities and larger spatial demands, they need more connected areas to maintain viable populations or to recover from stochastic extinctions due to insecticide use or their habitat drying up [139–141]. Consequently, habitat isolation negatively affects the abundance of many species of larval mosquito predators which can lead indirectly to an increase of mosquito densities [140, 142].

Irrigation schemes have been for a long time associated with an increase of mosquito populations by creating aquatic habitats such as paddy fields, irrigation canals and reservoirs [135, 143]. Irrigation practices have been investigated and intermittent irrigation has been identified as a method for limiting the development of mosquito larvae, save water and increase yields. Its basic concept within the vector control strategies is to interrupt the reproductive cycle of the mosquito by withholding water on a periodic basis from the rice fields. Drying rice fields for 2–6 days induces a high larval mortality and decreased adult density. However, the results are mitigated, some nice successes lead to a complete interruption of larval development, a decrease of parasite prevalence and an increase of rice yields, some failures include extreme cases of adult density increases (see [144] for a review). Failures have been attributed to invertebrate community disturbance with mosquitoes recovering faster than their predators. The difficulty to achieve the desired dry conditions was also put forth and was due to residual water collections such as footprints which never dried-up and allowed a concentration of mosquito larvae [134, 145, 146].

Urban areas are known to have a lower level of malaria endemicity than rural areas. This is assumed to be partly due to fewer *Anopheles* aquatic habitats which are frequently polluted and thus theoretically not favorable to larval development. However, the rapid growth of urban areas and the necessity to feed city populations increases urban and peri-urban agricultural irrigated sites which create larval aquatic habitats at the origin of most malaria cases [35, 147]. Consequently, urban areas concentrate issues regarding the use of chemical pesticides and pollutants, land and irrigation managements. All these factors are susceptible to impede predator colonization and indirectly promote the development of *Anopheles* species by creating enemy-free spaces. *Anopheles* species also show a high level of phenotypic plasticity and, in some localities, urban polluted waters are not a constraint to their survival anymore [148–152]. Moreover, these polluted breeding sites are frequently poorly structured (no plants), temporal and isolated. Together, these factors prevent the progression of biological successions and prohibit aquatic predator colonization [136].

Human interventions, instead of attempting to suppress *Anopheles* aquatic habitats, which is very difficult in most areas, should favor actions improving predator diversity and density. There have been many attempts to use predators as biological control agents, but few have shown satisfying results on adult or larval density reduction (but see [153] for a review). Moreover, their efficiency was generally evaluated through surveys of larval densities and/or emergence rates in treated water reservoirs, but adult entomological surveys or epidemiological follow-ups were not performed, impeding a real evaluation of their efficiency at reducing malaria transmission. The lack of efficiency could be due to both the way predator potential is evaluated and the intrinsic characteristics of predators. Indeed, first, predator potential as a biological agent is frequently assessed in laboratory studies with simple experimental designs in which predators are starved to standardize their “appetite” and the number of larvae killed serves as reference for their efficiency. However, such data make no sense in the field where predator efficiency will depend on many different factors: its own density, the density of its prey, the presence of alternative prey or other predators, the size and type of reservoir, the synchrony of prey-predator cycles, the presence of shelters for prey and the predator’s distribution in all the water reservoirs of the selected area. All this non-comprehensive list of factors has an impact on the density of mosquitos that will emerge from water reservoirs. Studies directly performed in the field give, most of the time, only an evaluation of what could be the productivity of a given water reservoir in the presence of the studied predator because all parameters cannot be quantified due to the difficulties inherent to the field. Consequently, the epidemiological impact of the use of predators to control mosquito populations can only be assessed in large trials along with epidemiological surveys. Secondly, a predator used as a biological agent has to be: a good self-disperser to reach all the breeding pools used by the mosquitoes; able to resist to the absence of mosquito larvae for long periods of time, either by entering dormancy or by feeding on alternative prey even though it has to show a strong preference for mosquito larvae; easy and cheap to rear (mass production, storage), easy to handle (release) and endure transport; should not be dangerous for the environment by creating a disequilibrium in the ecosystems in which it is introduced; should have a high reproductive capacity to limit rearing and release efforts over time ([33, 38], reviewed in [153, 154]). Unfortunately, few of the potential candidates meet all these criteria.

It is worth noting that across this review, we have had to digress toward other mosquito species due to the lack of data regarding *Anopheles*. Even among other mosquito taxa, few data are available on the real impact of predation on disease transmission. This highlights gaps in the knowledge of anopheline bio-ecology and other mosquito vectors and may constitute many research avenues to work on in the future. Among them, researches could focus especially on the following topics:

- As stressors are different in permanent and temporary reservoirs, and because colonization and the development of the abilities of predators are different from those of mosquitoes, it would be useful to lead further investigations on the predator community according to the size of the reservoir, especially in very small reservoirs (< 1 liter and about 10 days duration) such as footprints or phytothelmata which can be very productive in terms of mosquitoes but for which few data are available regarding predators.
- Mosquito predator communities in running water were also deeply neglected and need to be investigated to determine the type of pressure they exert on mosquito larvae and their impact on pathogen transmission.
- Any specific intervention on larval habitat (targeting or not predators) should include a follow-up of both predator diversity and densities. In the same way, both mosquito entomological surveys and pathogen epidemiological surveys should assess the sustainability of the intervention and the real impact of the intervention on pathogen transmission, respectively.
- Since testing and replicating all the potential experimental conditions and their combinations in both laboratory and field conditions is often time consuming, expensive and complicated to implement, modeling constitutes a strong potential alternative strategy to investigate the stressor effects. Moreover, as larval stress on pathogen transmission can be indirect, counter-intuitive and density- or species-dependent [12, 13, 155], modeling should allow better predictions on such impacts and could help to identify stressors that have synergetic or antagonistic effects.
- Finally, as rural populations can use pond water as drinking water for themselves and for animals, preservation of water quality by using natural predators, competitors or pathogens of mosquito larvae should be favored instead of the use of chemical insecticides. The benefits and drawbacks of using such control methods should be investigated with a One Health approach.

## Conclusions

The increase of insecticide resistance has made it necessary to design new tools or improve existing methods to better control vector-borne diseases. In a context of environmental crisis and global changes, environmentally friendly methods should be encouraged. In most cases, the sole use of predators to control malaria mosquitoes is obviously insufficient. Nevertheless, a growing evidence of their direct and indirect effects on vector life-history traits and consequently on mosquito vectorial capacity and pathogen transmission was recently highlighted [6, 8, 12, 83, 101, 102] triggering a new interest in predation stress mechanisms in mosquitoes, and more broadly onto the effects of environmental components for malaria vectors and malaria transmission [156]. Effects of the introduction of predators as control agents on vector populations in the wild and on malaria transmission are difficult to predict because of the environmental conditions which are much more complex than in a laboratory. However, human intervention intending to favor colonization and preservation of natural aquatic predators could be enhanced by preserving natural habitats and favoring permanent reservoirs which are known to shelter higher predator densities than small and ephemeral reservoirs [53, 145]. Preservation of the predators’ habitats could be part of sustainable and integrated vector-management strategies and could be a key component that helps to reduce both vector densities and malaria transmission through the enhancement of ecosystem services provided by predators or mosquito pathogens.
